# Using bibliometrics to evaluate outcomes and influence of translational biomedical research centers

**DOI:** 10.1017/cts.2021.863

**Published:** 2021-10-07

**Authors:** Kristine M. Bragg, Gwen C. Marchand, Jonathan C. Hilpert, Jeffrey L. Cummings

**Affiliations:** 1 Department of Educational Psychology and Higher Education, College of Education, University of Nevada, Las Vegas, Las Vegas, NV, USA; 2 Chambers-Grundy Center for Transformative Neuroscience, Department of Brain Health, School of Integrated Health Sciences, University of Nevada, Las Vegas, Las Vegas, NV, USA

**Keywords:** Research, social network analysis, bibliometrics, science of team science, Alzheimer’s disease, NIGMS, COBRE

## Abstract

**Introduction:**

Federal grant funding to support infrastructure development of translational biomedical research centers is a form of public health intervention. Establishing rigorous methods for measuring center success and outcomes is essential to justify continued funding.

**Methods:**

Bibliometric data compiled from a 5-year funding cycle of neurodegeneration and translational neuroscience research center were analyzed using the package bibliometrix for open-source software R and the NIH-developed research tool iCite.

**Results:**

The research team and their collaborators (n = 485) produced 157 grant-citing publications from 2015–2020. The science was produced by small research teams clustered around three main communities of topics: Alzheimer’s Disease, brain imaging, and neuropsychological testing in the elderly. Using the relative citation ratio, the publications produced by the research team were found to be influential when compared to other R01-funded publications.

**Conclusion:**

Recent developments in bibliometric analysis expand beyond traditional measurement capabilities to better understand the characteristics, outcomes, and influences of research teams. These findings can be used to inform researchers and institutions about research team composition, productivity, and success. Measures of research influence may be used to justify return on investment to funders.

## Introduction

The investment of federal extramural grant funds to create infrastructure for translational, collaborative, and interdisciplinary biomedical research is a form of public health intervention. Infrastructure development grants are an emerging cornerstone of funding agencies such as the National Institutes of Health (NIH), which invest a portion of their $32 billion annual biomedical funding portfolio into establishing and supporting institutes and centers [1]. In return, funded research centers must present trustworthy measures of their scientific contributions to assess productivity and justify continued infrastructure investment.

The primary mission of research infrastructure is to enable science. We adopt the perspective that evaluation of the infrastructure intervention be based on fit-for-purpose methodologies that tackle the complexities of real-world research translation [2]. We begin by reviewing several traditional bibliometric approaches for assessing publication trends. We then describe more sophisticated measures for evaluating collaborative scientific productivity and influence of publications stemming from grant-funded biomedical research institutes and centers over short-term and long-term horizons.

Taking a science of team science (SciTS) perspective, we identify methods to evaluate research center success and provide practical application of their use with data from an NIH-funded translational research team, the Center for Neurodegeneration and Translational Neuroscience (CNTN). The CNTN is funded by a Center of Biomedical Excellence (COBRE) from the National Institute of General Medical Sciences (NIGMS).

### Science of Team Science

Research teams, rather than sole authors, are increasingly responsible for the production of highly impacted and highly cited science [3–5]. In response to this shift, the emergent field of SciTS studies the characteristics and impacts of collaborative research teams. More specifically, SciTS studies structures, processes, and products associated with scientific teams spanning their conception, scientific discoveries, and eventual translation to clinical practice and public policy [6]. An effective team science collaboration is “expected to combine specialized expertise, theoretical approaches, and research methods across disciplinary boundaries, solving…complex problems and producing high-impact science” [7]. Scientists working in transdisciplinary collaborative teams must navigate intrapersonal, interpersonal, physical environment, organizational, societal and political, and technological factors in the pursuit of producing transformative science [8].

Evaluation of team science processes and outcomes may focus on the characteristics of teams such as developing culture and climate, management approaches, and determining optimal team size; they may also focus on traditional indicators of funding success such as the number and quality of scholarly products and traditional bibliometrics such as journal impact factors [9,10]. Recently, more sophisticated bibliometrics have become available that can give researchers and evaluators insights into the characteristics of scientific teams working within large-scale, grant-funded infrastructure interventions.

### Infrastructure as Intervention

The NIGMS awards funding to establish COBREs in states with historically low grant funding from the NIH through the Institutional Development Award (IDeA) program. Through the COBRE initiative, universities and independent research institutes/medical centers receive financial support to establish thematic multidisciplinary centers for biomedical research [11]. In this paper, we examine the publication output of the CNTN, a center funded to promote collaborative team science to enhance the biomedical and scientific workforce in Nevada (www.nevadacntn.org). The CNTN sponsors research projects to advance early-career investigators in the area of neurodegenerative disease and generates a repertoire of biomarker data stored in a collaborative database.

Funding is awarded in five-year cycles and is available for up to three phases, or 15 years, of total funding intervention. Additional phases of funding are awarded based on achieving a variety of center outcomes related to procurement of research dollars, technological innovation, and scholarly productivity and advancement. For the current study, we focus on scholarly productivity and advancement in the form of research influence via scholarly publications. Justification of publication output is crucial to securing funding for future phases, which ultimately leads to establishment of a self-sustained research center. In the following sections, we briefly review several common bibliometrics used to examine scholarly productivity, as well as describe a metric developed by the NIH, the relative citation ratio (RCR).

### Bibliometrics

Bibliometrics refers to the methods that utilize quantitative and statistical approaches to study various aspects of a chosen pool of publications [12,13]. Bibliometrics are measures of research articles or individual author’s influence or impact on future research. Common metrics included in bibliometric analyses are citations, journal impact factors (JIFs), scoring systems, and altmetrics [14]. More recently, new advances in machine learning, data mining, and networks science are broadening the field [15]. Because each type of metric delivers its own values as well as limitations, there is no general consensus on a “gold standard” metric of choice. Rather, widespread agreement suggests that a combination of metrics provides the strongest evaluation of productivity trends. Additionally, inclusion of data sources such as surveys and interviews allows for further triangulation and a broader view of collaboration [16].

#### Citations

Citation analysis is the most common bibliometric technique for measuring output of interdisciplinary teams and is widely considered at the heart of measuring scientific influence [17,18]. Citation analysis occurs in several forms such as straight citation counts or considers other value indicators such as co-citations, field-specific citations, or journal sources. Gathering information to conduct citation analysis is readily accessible through databases such as PubMed (National Center for Biotechnology Information), Web of Science (Clarivate Analytics), Scopus (Elsevier), and others.

Citation counts are the most traditional form of citation analysis, but as a standalone metric have often been of limited value. Citation counts do not factor in length of time since publication, scientific field, or topic. Without a formula for normalization, comparing citation counts from one article to another is more likely to be an “apples to oranges” rather than an “apples to apples” comparison because time is not treated as a confounding factor [14].

A co-citation is the frequency with which two documents are cited together by a later document [19]. Co-citation analysis can be carried out using networks of papers linked to an article of interest or reference article (RA). In the literature, three types of article-linked citation networks have been described. A co-citation network consists of other papers appearing in the reference lists alongside the RA, a citing network is the collection of papers citing the RA, and a cited-by network is the collection of papers in the reference list of the RA [20].

More sophisticated approaches to citation analysis involve the use of networks. Bibliometric networks use defined fields to study either structural relations or transaction relations between the chosen criteria [21]. An example of a structural relation would be between authors and their institutions, concept markers such as keywords, knowledge bases (cited-by articles), or knowledge users (citing articles). Transaction relations, such as flows of knowledge, may also be measured by citations and co-citations. The more documents that have co-cited the two papers, the stronger the relationship. Co-citation patterns can represent transactional relationships such as shared ideas, concepts, or methods [22]. They may also represent structural relationships between the authors, institutions, or journals publishing the article.

Networks are commonly reported using descriptive measures such as nodes, edges, density, transitivity, and path length [23–25]. Nodes represent each occurrence of the chosen criteria, and edges are ties between nodes. For example, in a keyword network, each keyword is a node, and keywords occurring together across publications receive an edge between them. Network density is the proportion of present edges from all possible edges in the network and is a measure of how well-connected a network is [24]. Transitivity measures small communities of nodes tied together in groups of three (triads) and is calculated as the number of observed transitive triads divided by the number of potential transitive triads [25]. Finally, a path is defined as the edges taken when going from one node to another. Often, multiple paths exist between any two nodes and the shortest path length is the path that involves the fewest steps [23,24].

#### Journal Impact Factor

The JIF is a measure of the downstream citations the average article in a journal receives [14]. The common interpretation is that the JIF is an indicator of journal quality, though its intended purpose was to help librarians make decisions about journals [26]. A recent critique highlighted two main fallacies of the JIF [27]. First, it is implausible that a high-quality paper can come only from a high-quality journal (deductive fallacy). Second, the mean number of citations articles in a journal receive during the initial years after publication does not equate to research quality (inductive fallacy). Despite long-standing and widespread criticism, the JIF continues to influence decision-making across academic and research-funding institutions [28].

#### The h-index

The h-index was developed as a way to quantify the scientific output of an individual researcher based on citation counts [29]. Noted strengths of the h-index are that it offers a more broadly balanced view of an individual author’s impact over simpler methods such as citation or publication counts [30]. Potential weaknesses of the h-index are that it is dependent on the author’s research field, susceptible to influence by self-citations, does not consider multi-authorship, is favorable to more established scientists, can never decrease, and has minimal sensitivity to highly cited papers [31]. Further, the h-index uses arbitrary defining parameters and provides inconsistent rankings for similarly performing scientists [32].

#### Relative Citation Ratio

In response to the numerous criticisms of commonly used metrics such as the JIF and h-index, new approaches for evaluating research output continue to be developed. Recently, the NIH supported development of the RCR, a time-sensitive metric which uses an article’s co-citation network to field-normalize the number of citations it has received [20]. An article’s co-citation network consists of all other articles it was *cited with* during each instance of the article being *cited by* another publication. The RCR compares the analyzed article’s citations per year with citations per year received by other NIH-funded articles in the same field and year. Other proposed strengths of the RCR include scalability from small to large portfolios and correlation with expert opinion.

An additional benefit of using the RCR to measure citation influence is the ability to analyze publications in groups rather than by author or by individual publication. The open-access NIH iCite tool (https://icite.od.nih.gov) reads in lists of PMIDs and provides an interactive dashboard displaying influence, translation, and citation data for the listed portfolio: influence is measured by the RCR, translation tracks and predicts translation of scientific knowledge to clinical studies, and citation displays open link-level citation metadata. Across the dashboard, data can be filtered by year and article type with the ability to toggle individual articles on or off. Report tables may be downloaded for future use.

### Study Aims

In this paper, we use bibliometrics to explore how infrastructure support for a multi-site translational research team contributes to scholarly outcomes using advanced network-based methods. We review two methods for examining trends of center-generated publications using the bibliometrix R-package and the NIH iCite tool. We accomplish our study goals through the following three aims:

Aim 1: Describe a multi-tiered approach for compiling a comprehensive list of grant citing publications to prepare them for bibliometric analysis.

Aim 2: Calculate and interpret measures of collaboration and productivity outcomes using bibliometrix analysis.

Aim 3: Calculate and interpret the utility of NIH iCite metrics to evaluate team success.

## Methods

### Study Aim 1: Data Compilation

We used a multi-phase approach to compile a comprehensive list of all scholarly products (journal articles, book chapters, conference papers, etc.) which cited the grant as a funding source between the years 2015–2020. First, we performed a product search using the grant number on the Web of Science and Scopus databases. Next, publication records reported by grant personnel, as part of the official reporting process, were exported from a Research Electronic Data Capture (REDCap) database [33]. Publication lists produced by each of the three sources were merged (akin to a full outer join) to generate a final, complete list. Any publications missing from the initial database searches were added manually to be able to export the complete list.

### Study Aim 2: Bibliometrix Analysis

Initial bibliometric analysis was carried out using the package bibliometrix through open-source software R [34]. The bibliometrix program specializes in science mapping by building data matrices for co-citation, coupling, scientific collaboration analysis, and co-word analysis. A co-citation connection is established by authors citing the articles of interest, whereas bibliographic coupling analyzes relationships among the articles of interest. A scientific collaboration network is a network where nodes are authors and links are co-authorships, and a co-occurrence network is used to map and cluster terms extracted from keywords, titles, or abstracts [34].

Bibliometrix reads in data extracted from a possible six main bibliographic databases: SCOPUS, Clarivate Analytics Web of Science, Digital Science Dimensions, The Lens, Cochrane Database of Systematic Reviews (CDSR), and PubMed/Medline. Data are extracted in different formats depending on the choice of database, with some formats affording analyses others may not. For example, a BibTeX (.bibtex) file exported from Scopus or Web of Science can be used for identifying citation, bibliographic coupling, and co-citation links between items while a PubMed file cannot [34].

In order to use co-citation data, the BibTeX format was selected for the analysis. The exported file contained citation metadata such as author(s), title, journal, date, PubMed Identification (PMID), author affiliations, article abstract, keywords, funding details, and references. Prior to the analysis, the BibTeX file was cleaned by editing author names to ensure that a single author was not counted as multiple authors due to different iterations of their name being used during the publishing process.

### Study Aim 3: iCite Analysis

A second bibliometric analysis was selected to measure article influence through the use of *iCite*, the publicly available research tool developed by the NIH. To run the analysis, a list of the PMIDs for all scholarly products identified in the data compilation process was uploaded. iCite calculates article influence through the previously described RCR. The benchmarking score of 1.0 indicates that an article is performing at the same level as the average NIH paper in its field for the same publication year. An article receiving a score of 2.0 would be interpreted as performing twice as well as its peers.

## Results

### Study Aim 1: Data Compilation

Discrepancies across all three sources used to compile a complete list of grant-citing products indicated that there is no single source that can be relied upon for a fully accurate list. The results returned the following: The Web of Science search returned 126 documents, the Scopus search returned 121 documents, and the REDCap database returned 124 documents. After a thorough inspection by combining all lists, deleting duplicates and other documents which were rendered invalid for their search terms, the total was 157 documents citing the grant. This means 31 grant-citing documents were missing from the Web of Science search and 36 were missing from the Scopus search.

To determine which database would be used for the bibliometric analysis, a final manual search for the missing documents in Web of Science and Scopus was performed by adding their PMIDs to the search terms. With this method, only eight missing documents were located by Web of Science, while 33 additional documents were located by Scopus. Therefore, Scopus was used to run the analyses.

### Study Aim 2: Bibliometrix Analysis

#### Descriptive Bibliometrics

The final Scopus output of 154 documents was the most comprehensive list available for BibTeX export to be used in the bibliometrix analysis. During the first five years and three months of funding, the research team produced 117 articles, 1 book chapter, 2 conference papers, 4 editorials, 1 published letter, 1 note, 26 review articles, and 2 short surveys which cited the funding number. Over time, the team produced an increased number of products with each additional year. The most notable jump occurred between 2017 and 2018 when the products almost doubled from 19 products reported in 2017 to 37 products in 2018. Small, but steady increases followed: 40 products in 2019, and 48 in 2020 (see Table 1).

**Table 1.** Descriptive bibliometrics for grant-citing documents for the years 2015–2020

Documents per year		Document type		Author information	
2015	1	Article	117	Authors	485
2016	9	Book chapter	1	Appearances	982
2017	19	Conference paper	2	Documents per author	0.32
2018	37	Editorial	4	Single author documents	13
2019	40	Letter/note	2	Multi-author documents	141
2020	48	Review	26	Co-authors per article	6.38
Total	154	Short survey	2	Collaboration index	3.43

Descriptive bibliometrics for 154 documents returned by Scopus and analyzed in R with package bibliometrix.

Author information showed 485 authors appeared 982 times producing 0.32 documents per author. This reflects the number of individual authors appearing in the queried document set, the summation of all authors per document, and the document total divided by the number of individual authors, respectively. Thirteen of the 154 documents contained a single author, the remaining 141 contained multiple authors. Per article, there was an average of 6.38 co-authors. Rather than calculating the ratio of total authors to total articles, the co-author per article index considers the total author appearances per actual article [23]. Finally, the collaboration index of 3.43 considered co-authors per article using only multi-authored articles. It is calculated as total authors of multi-authored articles divided by total multi-authored articles [35] (see Table 1).

#### Network Summaries

**Fig. 1.**
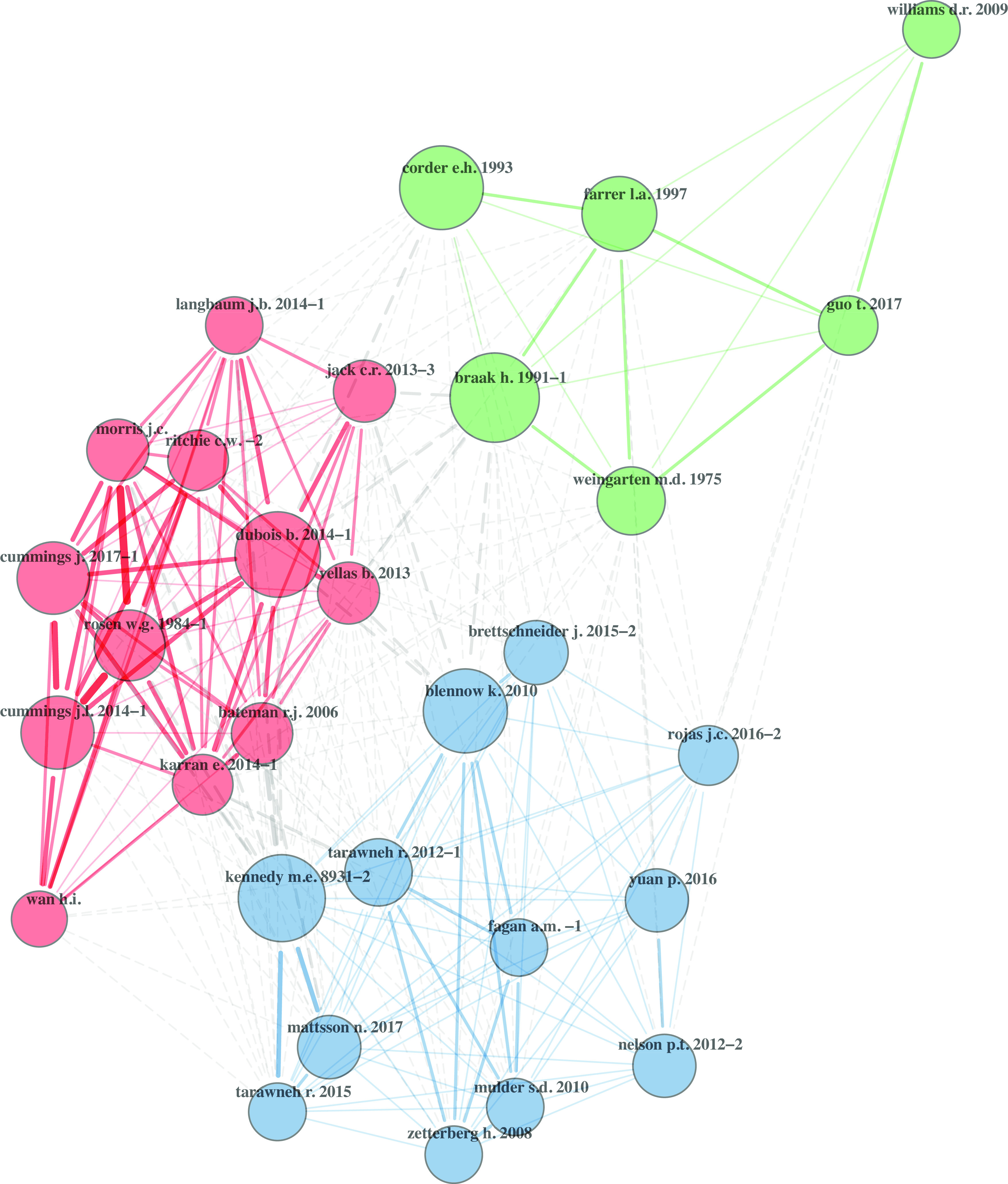
Cited-by network. Network self-organized into 3 citation communities. Node size is weighted by number of citations received. Main network statistics: size = 8325; density = 0.02; transitivity = 0.90; average path length = 3.43. Network compiled from 154 documents returned by Scopus. Figure produced in R with package bibliometrix.

**Fig. 2.**
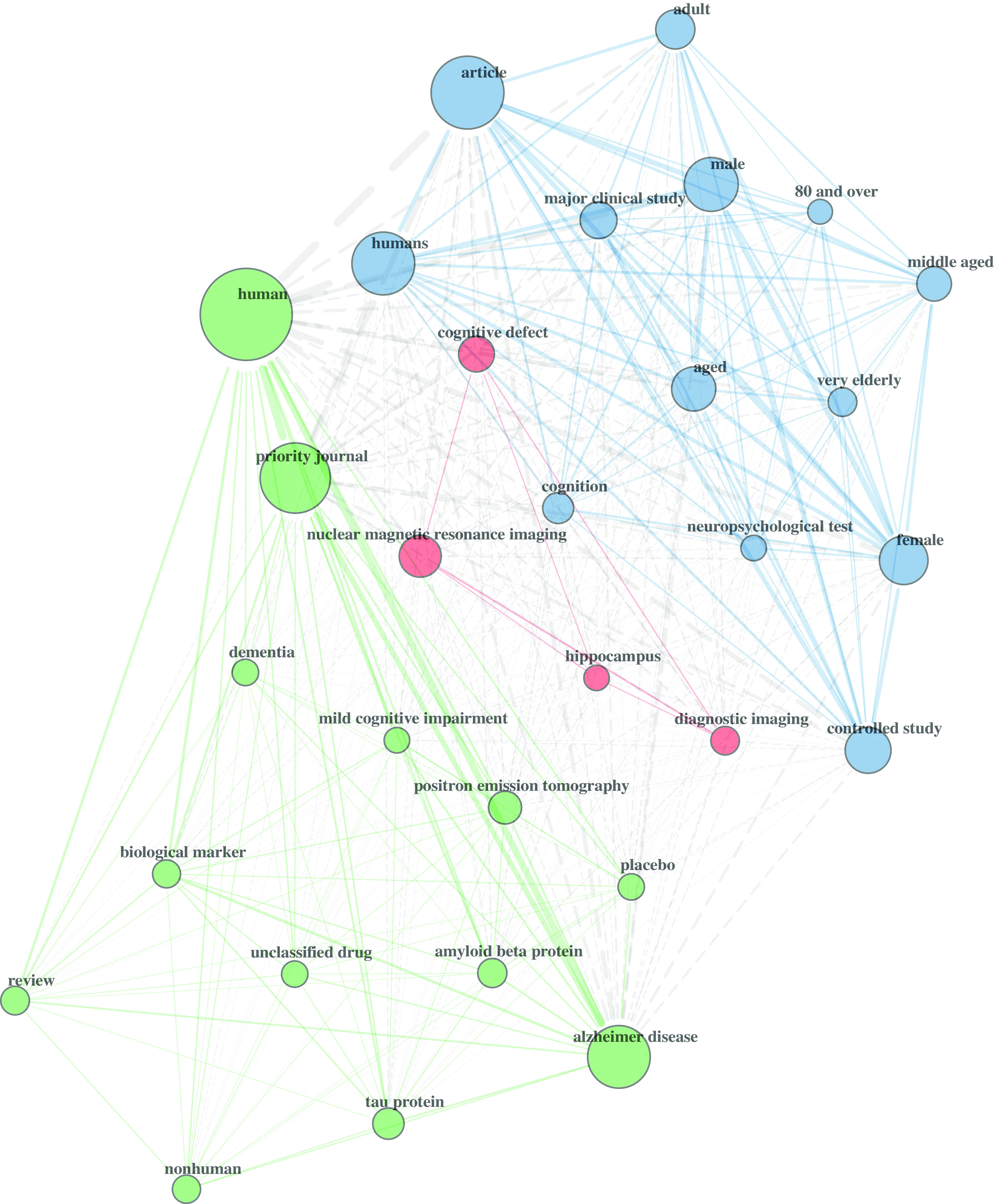
Keyword co-occurrences. Network self-organized into 3 keyword communities. Node size is weighted by number of keyword occurrences. Main network statistics: size = 485; density = 0.04, transitivity = 0.51, average path length = 2.47. Network compiled from 154 documents returned by Scopus for the top 30 keywords. Figure produced in R with package bibliometrix.

Visualizations are provided for the cited-by network (Fig. 1) and the keyword co-occurrences network (Fig. 2). The cited-by network (Fig. 1) is a network of all articles (n = 8325) cited by the research team. The network visualization displays three communities of frequently cited articles. Communities are considered as independent groups present in the network [36]. Larger circles (nodes) receive more weight (number of citations) in the network. The network exhibited low density (0.02), high transitivity (0.90), and moderate average path length (3.43).

The keyword co-occurrences (Fig. 2) display a network of the top 30 keyword choices (n = 485) used in the articles produced by the research team. Visual inspection of this network, like the cited-by network, reveals three communities of connected keywords. The network exhibited low density (0.04), moderate transitivity (0.51), and moderate average path length (2.47).

#### Network Analysis 2: iCite

The NIH iCite tool successfully read 152 documents from the list of PMIDs. A summary of the article influence metrics is provided in Table 2. The orientation of the analysis shifted to documents *citing* the uploaded list of products created by the research team. Per year, the research team’s articles received 4.71 citations (mean 4.71, SEM 1.5). This metric considers citations per full calendar year after publication, through the end of the records available to the NIH. For example, an article published in 2017 would be able to have citations counted for years 2018–2020 at the time of this inquiry. The total citations received over this time span divided by the years available equals the cites per year.

**Table 2.** iCite summary of article influence metrics

	Citations per year	RCR
Max	87.2	41.22
Mean	5.61	3.93
SEM	1.05	0.70
Median	1.50	1.44

Article influence metrics for 152 PMIDs accepted by iCite. Weighted relative citation ratio (RCR) of all articles = 469.38.

**Fig. 3.**
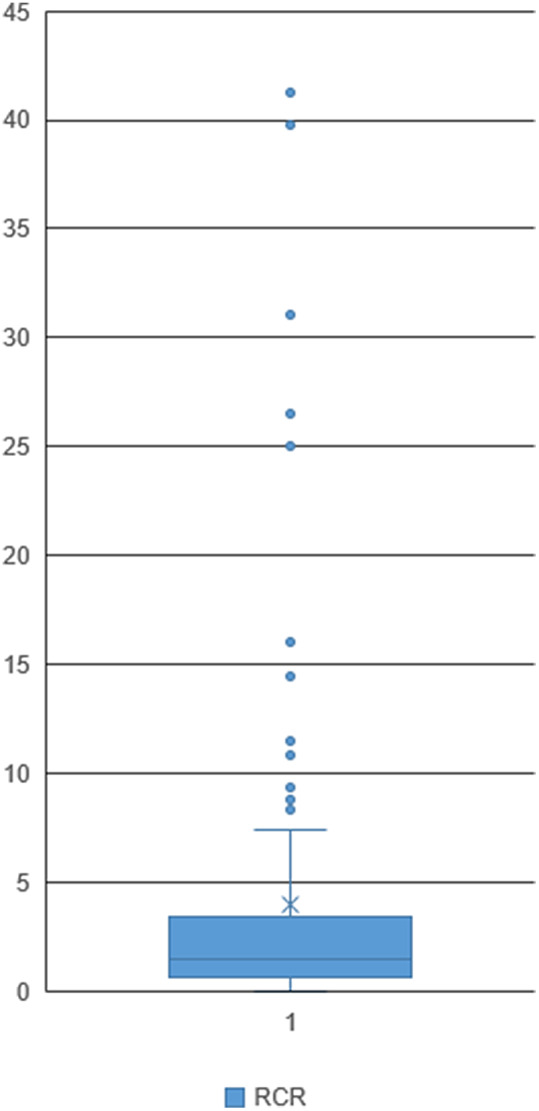
Relative citation ratio (RCR) box and whisker plot. The RCR distribution shows a clustering of products in the RCR range of 0.5–4 and a few articles covering the RCR range of approximately 8–42. Network compiled from 152 PMIDs accepted by iCite.

The product with the highest number of citations received 87.2 cites per year (max 87.2), while the median article cites per year was 1.50 (med 1.50). Descriptive statistics for the RCR are available in Table 2. As a measure of influence, the most influential article produced by the team received an RCR of 41.22 (max 41.22) with mean 4.71 (SEM 0.70). This means that the average product produced by this research team had performed, at the time of inquiry, nearly five times better than its field-normalized and time-normalized peer publications. A visualization of the RCR distribution displayed in Fig. 3 shows a clustering of products in the RCR range of 0.5–4 with a scattering of a dozen articles covering the RCR range of approximately 8–42.

## Discussion

In this study, we used two bibliometric analysis tools to explore how network approaches can be used within a SciTS evaluation framework to describe trends and characteristics of research collaboration and scientific knowledge production for an NIH-funded, multi-site translational research team. To satisfy Aim 1, we demonstrated that a multi-tiered approach was required to compile the most accurate list of all research products citing the grant. Neither self-report data, nor data obtained from two selected databases returned a list containing a complete, or even near complete, record of the products. This may be a challenge for interdisciplinary and translational teams, whose members may publish in discipline or field-specific journals that are indexed in different databases.

To meet Aim 2, the bibliometrix R-package provided descriptive measures of collaboration and productivity patterns. Values such as annual product output, product types, and author indices gave initial outcomes to characterize the data set. For the research team in the current analysis, a number of patterns were noted. First, the initial delay in annual product output suggests that two to three full years of funding were necessary to observe research output at full productivity. Due to the time it takes to establish working groups, produce research, and publish articles, bibliometric analysis may be useful over the life of a project to demonstrate the self-organization of collaborative groups around emergent scientific themes [25].

Next, in terms of collaboration reflected at the article level, the infrequent appearance of single-authored documents, in combination with the collaboration index, demonstrated that the products resulting from scientific activity were collaborative in nature. Specifically, an average of three to four-person teams produced each multi-authored paper. Existing SciTS literature has stated that co-authors are more heavily cited than single authors, an increase in authors leads to increased research impact, and increasing team size predicts research quality increases [4,5,37]. The average CNTN research team size is equal to the observed average team size across the landscape of science, engineering, and social sciences [5]. However, suggestions about optimal group size have not been a focus of team science research to date.

The bibliometrix analysis revealed nearly 500 authors have contributed to published products. In previous analyses conducted with this team of researchers, we reported that this COBRE research team consisted of 59 researchers in the third year of funding [25]. With that knowledge, we can conclude that a network of several hundred co-authors in and outside the center collaborated with the research team. This suggests that the funding provided to a small team of researchers contributed to scientific collaborations which extended far beyond the funded team members.

Turning to the network analyses, the cited-by network and keyword network demonstrated patterns of publication scope and content. The network statistics for both networks fell within expected ranges for non-random, self-organized networks [38–40], suggesting the presence of small world characteristic of a knowledge ecosystem [41]. The low density demonstrated that authors within this network collaborated with a select number of co-authors, which is logical given the large size of the network. The path length revealed that it takes an average of three steps for any one author within the network to reach any other author. Overall, this captured how information flows from the observed group of researchers throughout a larger scientific network. Members of the CNTN did not need to publish with large numbers of co-authors to be able to reach, within a few points of contact, a vast scientific community.

The cited-by network clustered around three distinct communities. This indicated that research team drew upon established science that is focused and cohesive. Similarly, the keyword co-occurrences formed three distinct communities. This demonstrated that the research team also produced cohesive research around three focused topics. The studies produced by the research team structured around human studies of Alzheimer’s disease, brain imaging, and neuropsychological testing in the elderly. This outcome showed that for this translational team, the research around humans dominated over animal models and can inform the principal investigator (PI) whether changes to the intervention should be made to shape research topics long term.

The NIH-developed iCite tool compared citations of the research team’s published documents against field-normalized and time-normalized studies available through the NIH database. While the analyses produced by bibliometrix provide information about the nature of the research team and scope of their products, the RCR allows for comparative evaluation of research success because it is capable of measuring influence of the research team against their research field(s) [19]. The RCR distribution for this research team demonstrates an overall high level of comparative success for the majority of the research articles. The dozen high performing outlier articles may additionally be highlighted by their investigators during annual reporting to the NIH.

A relatively new metric, the RCR, is beginning to be used as a comparative measure of research productivity across medicine, including translational research teams [42–44]. For our analysis, the RCR was used to indicate success for the CNTN as a whole. Additionally, the RCR can be filtered by author, year, and article type at both the article level and the group level. These are useful options to assess performance year to year, by research team designation, or for individual investigators.

Collectively, the results generated from the described network measures supply an array of information about productivity to be shared with the PI and advisory committees, whereas traditional assessment indices, such as JIF and h-index, attempt to communicate single indicators of impact, network outcomes provide an opportunity to conceptualize a number of complex factors that contribute to the dynamics of science creation. Network analyses also provide insights on research collaboration and productivity in absence of a control group or implied causal mechanism. Our current analyses presented publication output as a single capture, representative of 5 years of publishing efforts. Should a PI wish to observe patterns annually, individual data sets per year are another option.

Initial questions such as who published with whom, what types of research documents were produced, what size research teams contributed to products, and what research topics emerged provide a way to qualify the community structure for a given scientific team. As a first level of insight, these descriptors may inform the PI about the characteristics of the research team and the nature of their research output. Depicting patterns of productivity over time is also of value to detect areas of underperformance or realize untapped resources within the network. For example, a PI may wish to increase certain types of publication output or review the groupings of collaborative teams. The PI may also notice unexpected communities of research topics that could potentially drive future research design.

The PI and advisory committees must also justify quality of research produced to report back to the funding agency. Research quality can be assessed by the RCR as a measure of influence and also may be informed by the cited-by and keyword co-occurrences networks. When applied in-context by experts in the given field, the networks speak to the content of publications beyond what may be drawn from publication counts or debatably flawed impact indices.

Overall, the network analyses provide a cohesive and multi-dimensional approach distinct from other bibliometric methodology. Applying a combination of descriptive, comparative, and visually compelling results enhances the ability to tell the story of a collaborative research team. This allows the PI or other individual investigators to leverage different types of network information to secure additional funding in the form of continued institutional support or individual career development.

### Limitations

As an exploratory study situated in the still-developing fields of SciTS and bibliometrics, several limitations emerged throughout the process. First, performing a grant number search to locate scholarly products limited our ability to include products for which the authors may have forgotten to cite the funding source. However, in the case of the CNTN, project leads, core directors, and administrators were reminded frequently to cite the award and were additionally required to confirm funding acknowledgment status during bi-annual product reporting. Therefore, the grant number was expected to be a reliable search option for this research team.

Another consideration is that each available search tool has its limitations. For example, the iCite tool is only able to analyze publications that have been issued a PMID. Additionally, database searches will not only exclude valuable research products, such as conference presentations and abstracts, but also limit the assessment of outcomes or success to quantifiable conditions. The use of alternative analytical tools may yield different results.

## Conclusions

Developing and refining trustworthy and effective methods for assessing the outcomes of grant-funded biomedical translational research teams is an ongoing endeavor. Through the use of two publicly available bibliometric analysis tools, we were able to extend our analyses beyond traditional, simplistic views of publication trends to more rigorous use of citation metadata. Multi-dimensional network analysis helps create a more complete understanding of collaboration characteristics, research output content, and research influence. These approaches can be used by researchers, institutions, and funders to better understand the characteristics of successful research teams, determine what size and composition of research teams optimizes productivity, and assess the investment that produces the greatest productivity. Future studies should consider exploring additional metrics available through both included research tools to progress our collective understanding of how to define success for translational research teams.
